# The genome sequence of an ichneumonid wasp, *Ophion crassicornis* Brock, 1982 (Hymenoptera: Ichneumonidae)

**DOI:** 10.12688/wellcomeopenres.26848.1

**Published:** 2026-07-27

**Authors:** Gavin R. Broad, Chris Fletcher, Stephanie Holt, Laura Sivess, Inez Januszczak, Natural History Museum Genome Acquisition Lab, Darwin Tree of Life Barcoding Collective, Wellcome Sanger Institute Tree of Life Management, Samples and Laboratory team, Wellcome Sanger Institute Scientific Operations: Sequencing Operations, Wellcome Sanger Institute Tree of Life Core Informatics team, Tree of Life Core Informatics collective, Darwin Tree of Life Consortium

**Affiliations:** 1 Natural History Museum, London, England, UK

**Keywords:** Ophion crassicornis, ichneumonid wasp, parasitoid wasp, genome sequence, chromosomal, Hymenoptera

## Abstract

We present a genome assembly from an individual female *Ophion crassicornis* (ichneumonid wasp; Arthropoda; Insecta; Hymenoptera; Ichneumonidae). The genome sequence has a total length of 726.34 megabases. Most of the assembly (98.51%) is scaffolded into 13 chromosomal pseudomolecules. The mitochondrial genome has also been assembled, with a length of 38.03 kilobases. This assembly was generated as part of the Darwin Tree of Life project, which produces genomes for eukaryotic species found in Britain and Ireland.

## Species taxonomy

Eukaryota; Opisthokonta; Metazoa; Eumetazoa; Bilateria; Protostomia; Ecdysozoa; Panarthropoda; Arthropoda; Mandibulata; Pancrustacea; Altocrustacea; Allotriocarida; Hexapoda; Insecta; Dicondylia; Pterygota; Neoptera; Eumetabola; Endopterygota; Hymenoptera; Apocrita; Ichneumonoidea; Ichneumonidae; Ophioninae; Ophion group; *Ophion*; *Ophion crassicornis* Brock, 1982 (NCBI:txid1539394).

## Background

*Ophion crassicornis* is a nocturnal parasitoid wasp (family Ichneumonidae, subfamily Ophioninae). This is one of around 34 British *Ophion* species (Broad, unpublished), many very similar and several only described relatively recently ( Johansson & Cederberg, 2019). As with most of these species, *O. crassicornis* is almost entirely testaceous (pale reddish) with very long antennae, large eyes and ocelli. *Ophion crassicornis* was described in 1982 from British specimens ( Brock, 1982) and has since been found rather widely in Central Europe as far North as Sweden ( Johansson & Cederberg, 2019). Brock’s (1982) concept of *O. crassicornis* was quite broad when compared to current species concepts, based on a combination of DNA barcoding and morphology, with *O. angularis*, *O. borealis* and *O. broadi* all previously confused under the name, ‘ *O. crassicornis*’. Eight paratypes of *O. crassicornis* in the Natural History Museum have been reidentified by GRB as *O. angularis.*

One of several *Ophion* species with very long antennae (typically around 60 flagellar segments) and a distinct gap between the eye and lateral ocellus, *O. crassicornis* can best be identified by the shape of the epicnemial carina (not sharply angled ventrally, as in *O. angularis*), the comparatively wide space between the anterior tentorial pits on the head (compared to *O. borealis*) and the strongly arched anterior transverse carina of the propodeum (gently curved in *O. borealis*) ( Johansson & Cederberg, 2019). Additionally, *O. crassicornis* can often be recognized by the dark underside of the metasoma.

In Britain, *O. crassicornis* is widespread, although not known from northernmost Scotland and may be associated mainly with deciduous woodland, based on data assembled by GRB. The species is on the wing from mid-May to early July, most frequently collected in June. Although *O. crassicornis* is by no means rare, it is one of a small number of British *Ophion* with no reliable host records ( Shaw & Broad, 2023), other than a single specimen in the Natural History Museum reared from an unknown species of Noctuidae (Lepidoptera). The host record published by Brock (1982) and repeated by Johansson & Cederberg (2019), of *Aporophyla nigra* (Lepidoptera: Noctuidae), in fact refers to a rearing of *O. angularis* (specimen in the Natural History Museum).

The genomes of several species of *Ophion* have now been published, enabling comparative analyses of a group of parasitoids which are easily sampled with light traps and very amenable to resequencing projects.

## Methods

### Sample acquisition and DNA barcoding

**Figure 1.**
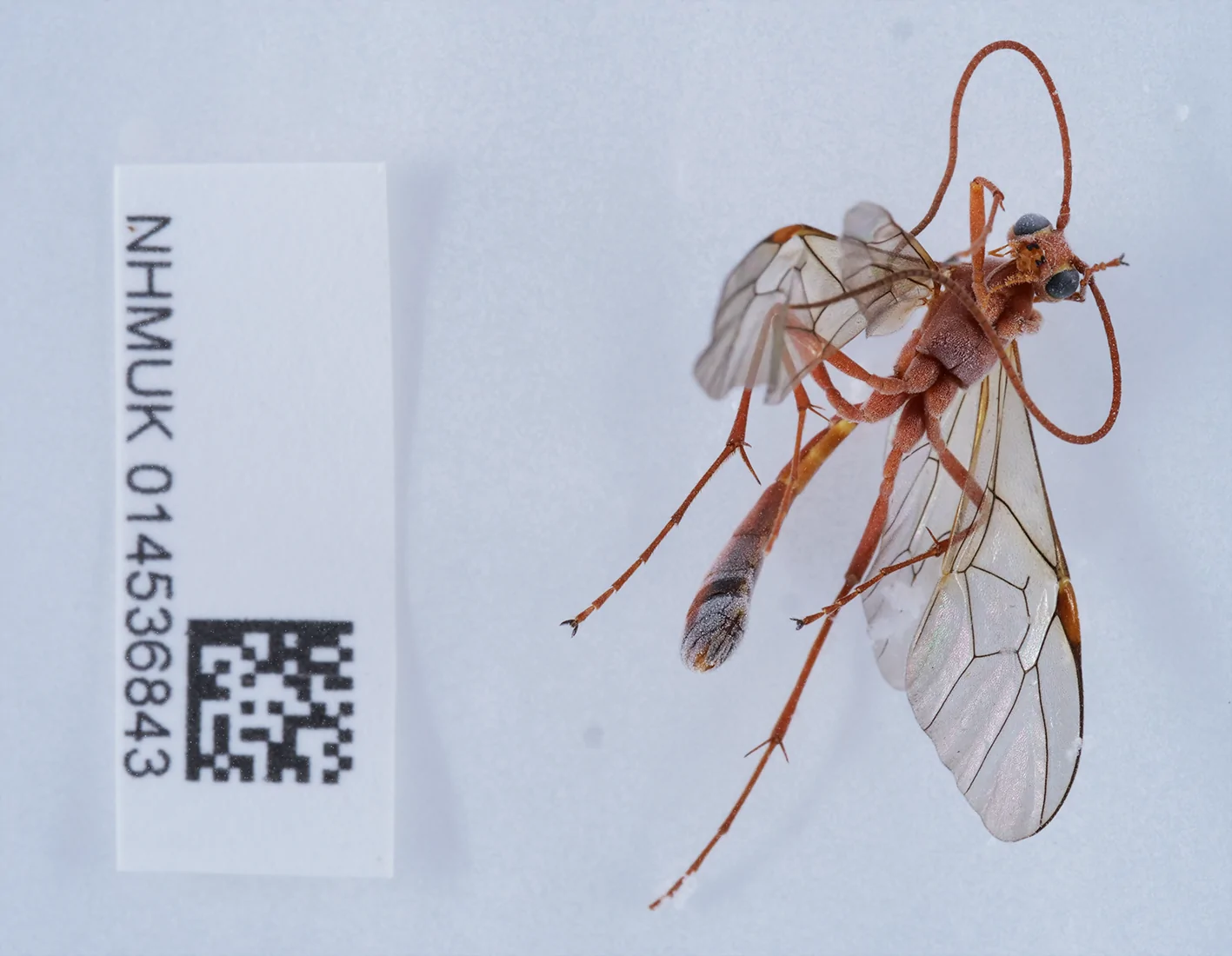
Photograph of the *Ophion crassicornis* (iyOphCras1) specimen used for genome sequencing.

The specimen of *Ophion crassicornis* used for genome sequencing (specimen ID NHMUK014536843, ToLID iyOphCras1; Figure 1) was collected from Gilbert White’s House, England, UK (latitude 51.09, longitude −0.94) on 2021-06-10. The specimen was collected by Chris Fletcher, Steph Holt, Laura Sivess, Gavin Broad and Inez Januszczak and identified by Gavin Broad. The iyOphCras1 specimen was also used for Hi-C and RNA sequencing.

The initial identification was verified by an additional DNA barcoding process according to the framework developed by Twyford *et al.* (2024). A small sample was dissected from the specimen and stored in ethanol, while the remaining parts were shipped on dry ice to the Wellcome Sanger Institute (WSI) (see the protocol). The tissue was lysed, the COI marker region was amplified by PCR, and amplicons were sequenced and compared to the BOLD database, confirming the species identification ( Crowley *et al.*, 2023). Following whole genome sequence generation, the relevant DNA barcode region was also used alongside the initial barcoding data for sample tracking at the WSI ( Twyford *et al.*, 2024). The standard operating procedures for Darwin Tree of Life barcoding are available on protocols.io.

### Nucleic acid extraction

Detailed protocols for nucleic acid extraction developed at the Wellcome Sanger Institute (WSI) Tree of Life Core Laboratory are available on protocols.io ( Howard *et al.*, 2025). The iyOphCras1 specimen was weighed and triaged to determine the appropriate extraction protocol. For HMW DNA extraction, 24 mg of tissue from the thorax was used. The tissue was homogenised by powermashing using a PowerMasher II tissue disruptor. High molecular weight (HMW) DNA was extracted using the Automated MagAttract v2 protocol. Sheared DNA was purified by manual SPRI (solid-phase reversible immobilisation). The concentration of the sheared and purified DNA was assessed using a Nanodrop spectrophotometer and Qubit Fluorometer using the Qubit dsDNA High Sensitivity Assay kit. Fragment size distribution was evaluated by running the sample on the FemtoPulse system. For this sample, the final post-shearing DNA had a Qubit concentration of 34.6 ng/μL and a yield of 1 591.60 ng, with a fragment size of 12.6 kb. The 260/280 spectrophotometric ratio was 1.87, and the 260/230 ratio was 1.78. The Genomic Quality Number (GQN) was 4.6.

RNA was extracted from abdomen tissue of the iyOphCras1 specimen in the Tree of Life Laboratory at the WSI using the RNA Extraction: Automated MagMax™ *mir*Vana protocol. The RNA concentration was assessed using a Nanodrop spectrophotometer and a Qubit Fluorometer using the Qubit RNA Broad-Range Assay kit. Analysis of the integrity of the RNA was done using the Agilent RNA 6000 Pico Kit and Eukaryotic Total RNA assay.

### PacBio HiFi library preparation and sequencing

Library preparation and sequencing were performed at the WSI Scientific Operations core. Libraries were prepared using the SMRTbell Prep Kit 3.0 (Pacific Biosciences, California, USA), following the manufacturer’s instructions. The kit includes reagents for end repair/A-tailing, adapter ligation, post-ligation SMRTbell bead clean-up, and nuclease treatment. Size selection and clean-up were performed using diluted AMPure PB beads (Pacific Biosciences). DNA concentration was quantified using a Qubit Fluorometer v4.0 (ThermoFisher Scientific) and the Qubit 1X dsDNA HS assay kit. Final library fragment size was assessed with the Agilent Femto Pulse Automated Pulsed Field CE Instrument (Agilent Technologies) using the gDNA 55 kb BAC analysis kit.

The sample was sequenced using the Sequel IIe system (Pacific Biosciences, California, USA). The concentration of the library loaded onto the Sequel IIe was in the range 40–135 pM. SMRT Link software, a PacBio web-based workflow manager, was used to set up and monitor the run, and to perform primary and secondary analysis of the data upon completion.

### Hi-C

***Sample preparation and crosslinking***

The Hi-C sample was prepared from 20–50 mg of frozen head tissue from the iyOphCras1 sample using the Arima-HiC v2 kit (Arima Genomics). Following the manufacturer’s instructions, tissue was fixed and DNA crosslinked using TC buffer to a final formaldehyde concentration of 2%. The tissue was homogenised using the Diagnocine Power Masher-II. Crosslinked DNA was digested with a restriction enzyme master mix, biotinylated, and ligated. Clean-up was performed with SPRISelect beads before library preparation. DNA concentration was measured with the Qubit Fluorometer (Thermo Fisher Scientific) and Qubit HS Assay Kit. The biotinylation percentage was estimated using the Arima-HiC v2 QC beads.

***Hi-C library preparation and sequencing***

Biotinylated DNA constructs were fragmented using a Covaris E220 sonicator and size selected to 400–600 bp using SPRISelect beads. DNA was enriched with Arima-HiC v2 kit Enrichment beads. End repair, A-tailing, and adapter ligation were carried out with the NEBNext Ultra II DNA Library Prep Kit (New England Biolabs), following a modified protocol where library preparation occurs while DNA remains bound to the Enrichment beads. Library amplification was performed using KAPA HiFi HotStart mix and a custom Unique Dual Index (UDI) barcode set (Integrated DNA Technologies). Depending on sample concentration and biotinylation percentage determined at the crosslinking stage, libraries were amplified with 10–16 PCR cycles. Post-PCR clean-up was performed with SPRISelect beads. Libraries were quantified using the AccuClear Ultra High Sensitivity dsDNA Standards Assay Kit (Biotium) and a FLUOstar Omega plate reader (BMG Labtech).

Prior to sequencing, libraries were normalised to 10 ng/μL. Normalised libraries were quantified again to create equimolar and/or weighted 2.8 nM pools. Pool concentrations were checked using the Agilent 4200 TapeStation (Agilent) with High Sensitivity D500 reagents before sequencing. Sequencing was performed using paired-end 150 bp reads on the Illumina NovaSeq 6000.

### RNA library preparation and sequencing

Libraries were prepared using the NEBNext ^®^ Ultra II Directional RNA Library Prep Kit for Illumina (New England Biolabs), following the manufacturer’s instructions. Poly(A) mRNA was isolated from 100 ng of total RNA using oligo (dT) beads, converted to cDNA, and uniquely indexed; 14 PCR cycles were performed. Libraries were prepared with an intended fragment size of 100–300 bp. Libraries were quantified, normalised, and pooled equimolarly to a final concentration of 2.8 nM before dilution to 150 pM for loading. Sequencing was carried out on the Illumina NovaSeq 6000, generating paired-end reads.

### Genome assembly

Prior to assembly of the PacBio HiFi reads, a database of *k*-mer counts ( *k* = 31) was generated from the filtered reads using FastK. GenomeScope2 ( Ranallo-Benavidez *et al.*, 2020) was used to analyse the *k*-mer frequency distributions, providing estimates of genome size, heterozygosity, and repeat content.

The HiFi reads were assembled using Hifiasm ( Cheng *et al.*, 2021) with the --primary option. Haplotypic duplications were identified and removed using purge_dups ( Guan *et al.*, 2020). The Hi-C reads ( Rao *et al.*, 2014) were mapped to the primary contigs using bwa-mem2 ( Vasimuddin *et al.*, 2019), and the contigs were scaffolded in YaHS ( Zhou *et al.*, 2023) with the --break option for handling potential misassemblies. The scaffolded assemblies were evaluated using Gfastats ( Formenti *et al.*, 2022), BUSCO ( Manni *et al.*, 2021) and MerquryFK ( Rhie *et al.*, 2020).

The mitochondrial genome was assembled using OATK ( Zhou *et al.*, 2025).

### Assembly curation

The assembly was decontaminated using the Assembly Screen for Cobionts and Contaminants ( ASCC) pipeline. TreeVal was used to generate the flat files and maps for use in curation. Manual curation was conducted primarily in PretextView and HiGlass ( Kerpedjiev *et al.*, 2018). Scaffolds were visually inspected and corrected as described by Howe *et al*. (2021). Manual corrections included 200 breaks, 452 joins, and removal of 62 haplotypic duplications. This reduced the scaffold count by 35.2%, increased the scaffold N50 by 9.7%, and reduced the total assembly length by 0.6%. The curation process is described at https://gitlab.com/wtsi-grit/rapid-curation . PretextSnapshot was used to generate a Hi-C contact map of the final assembly.

### Assembly quality assessment

The MerquryFK tool ( Rhie *et al.*, 2020) was run in a Singularity container ( Kurtzer *et al.*, 2017) to evaluate *k*-mer completeness and assembly quality for the primary and alternate haplotypes using the *k*-mer database ( *k* = 31) computed prior to genome assembly. The analysis outputs included assembly QV scores and completeness statistics.

The genome was analysed using the BlobToolKit pipeline, a Nextflow implementation of the earlier Snakemake version ( Challis *et al.*, 2020). The pipeline aligns PacBio reads using minimap2 ( Li, 2018) and SAMtools ( Danecek *et al.*, 2021) to generate coverage tracks. It runs BUSCO ( Manni *et al.*, 2021) using lineages identified from the NCBI Taxonomy ( Schoch *et al.*, 2020). For the three domain-level lineages, BUSCO genes are aligned to the UniProt Reference Proteomes database ( Bateman *et al.*, 2023) using DIAMOND blastp ( Buchfink *et al.*, 2021). The genome is divided into chunks based on the density of BUSCO genes from the closest taxonomic lineage, and each chunk is aligned to the UniProt Reference Proteomes database with DIAMOND blastx. Sequences without hits are chunked using seqtk and aligned to the NT database with blastn ( Altschul *et al.*, 1990). The BlobToolKit suite consolidates all outputs into a blobdir for visualisation. The BlobToolKit pipeline was developed using nf-core tooling ( Ewels *et al.*, 2020) and MultiQC ( Ewels *et al.*, 2016), with containerisation through Docker ( Merkel, 2014) and Singularity ( Kurtzer *et al.*, 2017).

## Genome sequence report

### Sequence data

**Figure 2.**
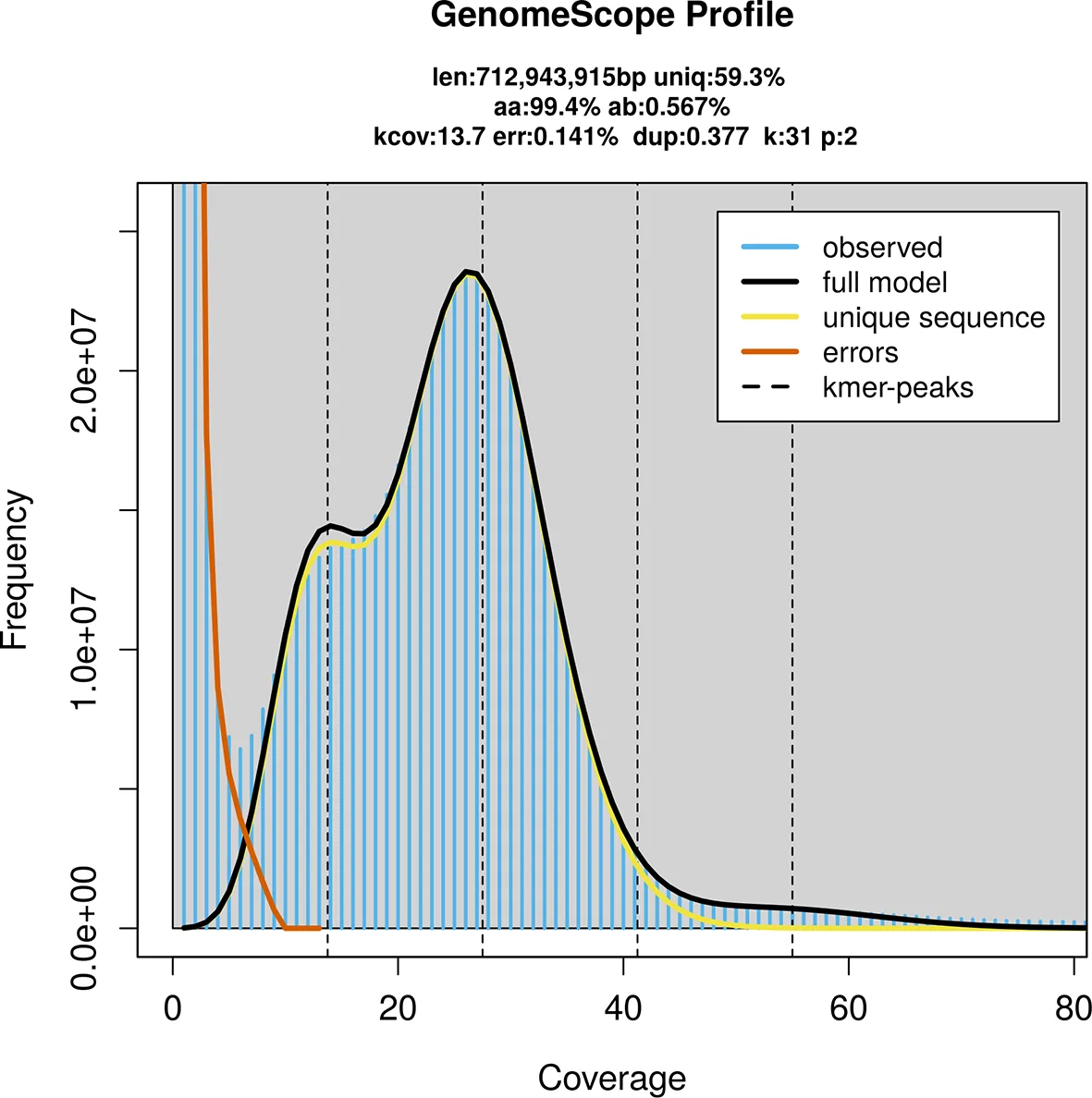
Frequency distribution of *k*-mers generated using GenomeScope2. The plot shows observed and modelled *k*-mer spectra, providing estimates of genome size, heterozygosity, and repeat content based on unassembled sequencing reads.

PacBio sequencing of the *Ophion crassicornis* specimen generated 20.56 Gb (gigabases) from 2.45 million reads, which were used to assemble the genome. GenomeScope2.0 analysis estimated the haploid genome size at 714.27 Mb, with a heterozygosity of 0.57% and repeat content of 40.66% ( Figure 2). These estimates guided expectations for the assembly. Based on the estimated genome size, the sequencing data provided approximately 28× coverage. Hi-C sequencing produced 130.14 Gb from 430.92 million reads, which were used to scaffold the assembly. RNA sequencing data were also generated and are available in public sequence repositories. Table 1 summarises the specimen and sequencing details.

**Table 1.** Specimen and sequencing data for *Ophion crassicornis* (BioProject PRJEB60661).

Platform	PacBio HiFi	Hi-C	RNA-seq
**ToLID**	iyOphCras1	iyOphCras1	iyOphCras1
**Specimen ID**	NHMUK014536843	NHMUK014536843	NHMUK014536843
**BioSample (source individual)**	SAMEA14448265	SAMEA14448265	SAMEA14448265
**BioSample (tissue)**	SAMEA14452961	SAMEA14452962	SAMEA14452960
**Tissue**	thorax	head	abdomen
**Instrument**	Sequel II	Illumina NovaSeq 6000	Illumina NovaSeq 6000
**Run accessions**	ERR11029678	ERR11040184	ERR11837477
**Read count total**	2.45 million reads	430.92 million read pairs	35.75 million read pairs
**Base count total**	20.56 Gb	130.14 Gb	10.80 Gb

### Assembly statistics

The primary haplotype was assembled, and contigs corresponding to an alternate haplotype were also deposited in INSDC databases. The final assembly has a total length of 726.34 Mb in 574 scaffolds, with 3 251 gaps, and a scaffold N50 of 55.84 Mb ( Table 2).

**Table 2.** Genome assembly data for *Ophion crassicornis.*

Genome assembly	Primary assembly
**Assembly name**	iyOphCras1.1
**Assembly accession**	GCA_982089845.1
**Alternate haplotype accession**	GCA_982093725.1
**Assembly level**	chromosome
**Span (Mb)**	726.34
**Number of chromosomes**	13
**Number of contigs**	3 825
**Contig N50**	0.37 Mb
**Number of scaffolds**	574
**Scaffold N50**	55.84 Mb
**Organelles**	Mitochondrial genome: 38.03 kb

**Figure 3.**
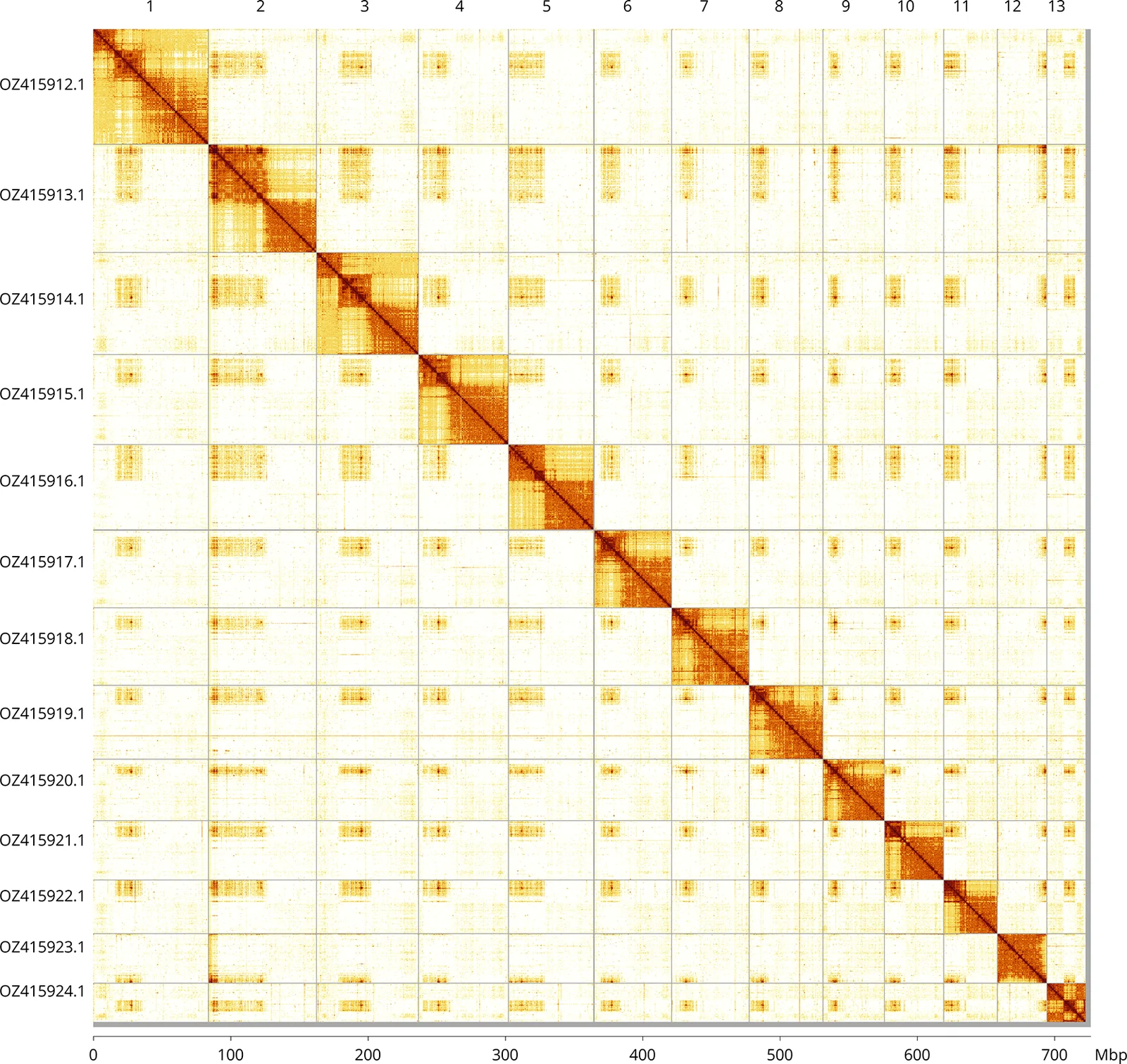
Hi-C contact map of the *Ophion crassicornis* genome assembly. Assembled chromosomes are shown in order of size and labelled along the axes, with a megabase scale shown below. The plot was generated using PretextSnapshot.

Most of the assembly sequence (98.51%) was assigned to 13 chromosomal-level scaffolds. These chromosome-level scaffolds, confirmed by Hi-C data, are named according to size ( Figure 3; Table 3).

**Table 3.** Chromosomal pseudomolecules in the primary genome assembly of *Ophion crassicornis* iyOphCras1.

INSDC accession	Molecule	Length (Mb)	GC%
OZ415912.1	1	83.02	38.50
OZ415913.1	2	77.82	39.50
OZ415914.1	3	73.57	38.50
OZ415915.1	4	64.88	38.50
OZ415916.1	5	61.82	39
OZ415917.1	6	55.84	38.50
OZ415918.1	7	55.79	38.50
OZ415919.1	8	53.30	38.50
OZ415920.1	9	44.17	38.50
OZ415921.1	10	42.75	39
OZ415922.1	11	38.67	39.50
OZ415923.1	12	35.65	38.50
OZ415924.1	13	28.25	38.50

The mitochondrial genome was also assembled (length 38.03 kb, OZ415925.1). This sequence is included as a contig in the multifasta file of the genome submission and as a standalone record.

### Assembly quality metrics

**Figure 4.**
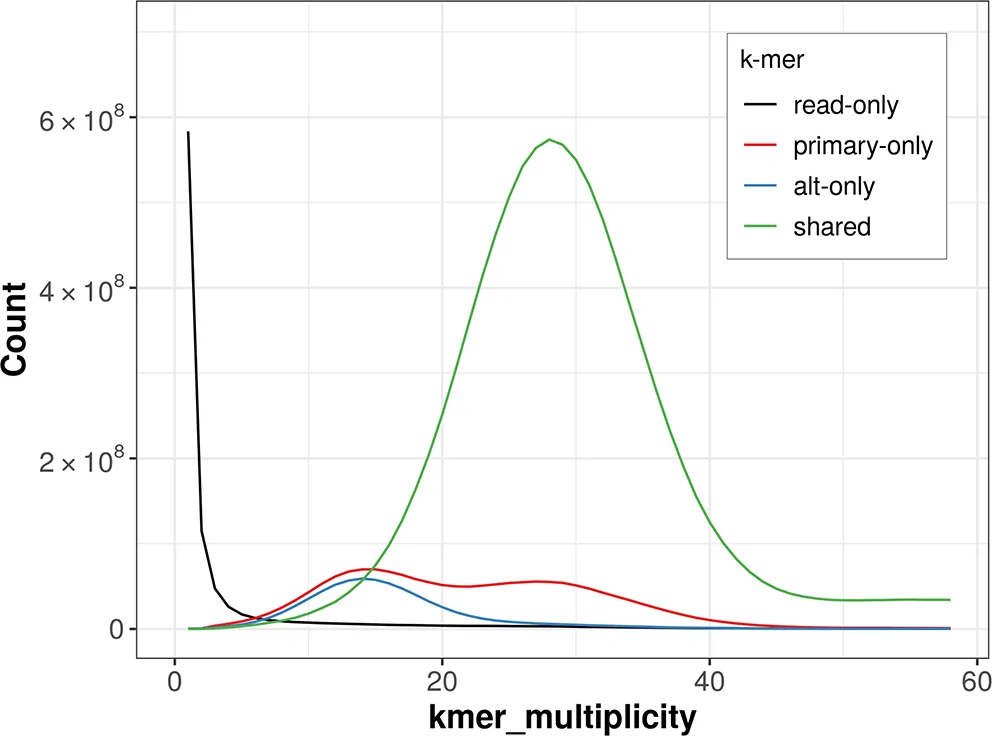
Evaluation of *k*-mer completeness using MerquryFK. This plot illustrates the recovery of *k*-mers from the original read data in the final assemblies. The horizontal axis represents *k*-mer multiplicity, and the vertical axis shows the number of *k*-mers. The black curve represents *k*-mers that appear in the reads but are not assembled. The green curve corresponds to *k*-mers shared by both haplotypes, and the red and blue curves show *k*-mers found only in one of the haplotypes.

The combined primary and alternate assemblies achieve an estimated QV of 55.8. The *k*-mer completeness is 89.10% for the primary assembly, 82.05% for the alternate haplotype, and 98.11% for the combined assemblies ( Figure 4).

**Figure 5.**
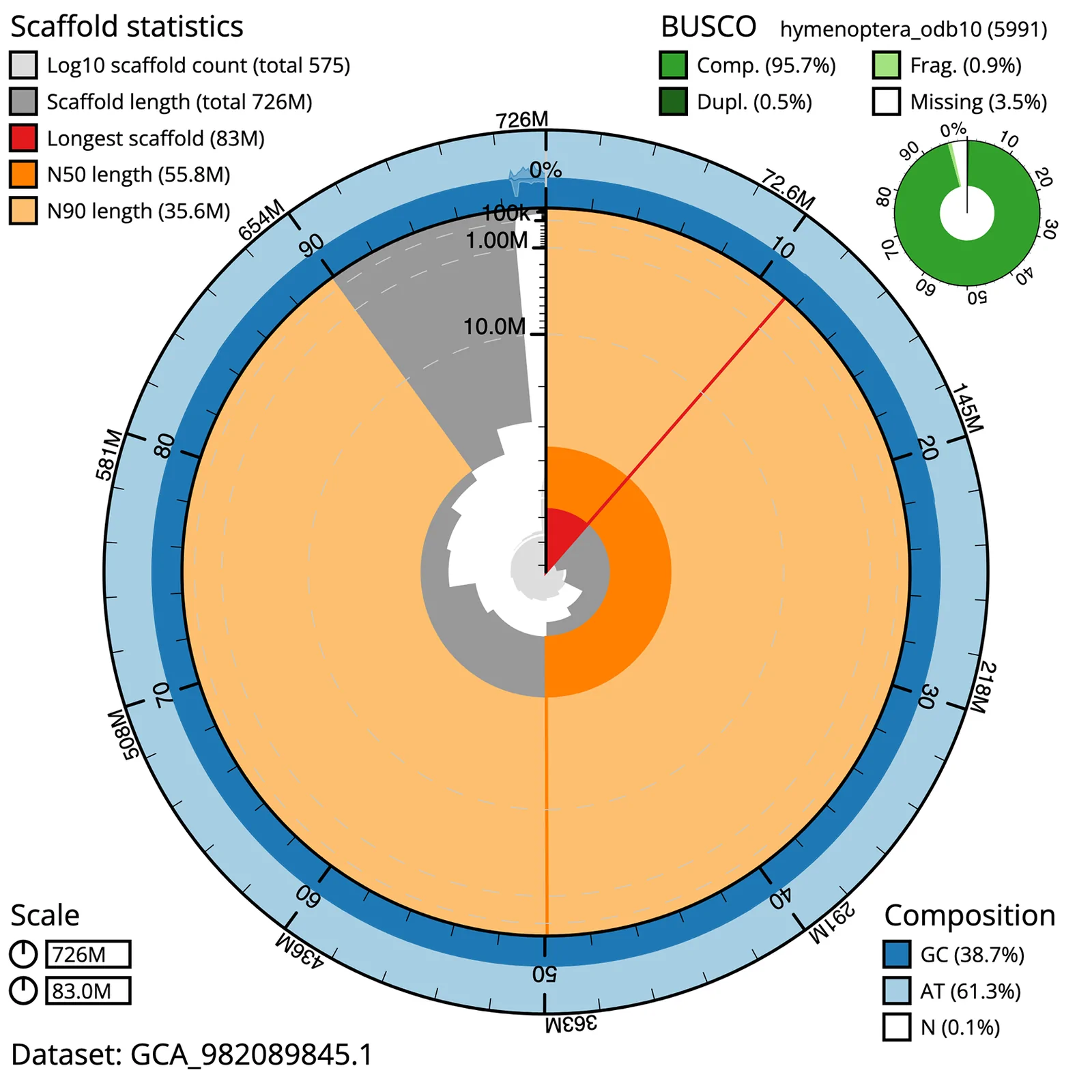
Assembly metrics for iyOphCras1.1. The BlobToolKit snail plot provides an overview of assembly metrics and BUSCO gene completeness. The circumference represents the length of the whole genome sequence, and the main plot is divided into 1 000 bins around the circumference. The outermost blue tracks display the distribution of GC, AT, and N percentages across the bins. Scaffolds are arranged clockwise from longest to shortest and are depicted in dark grey. The longest scaffold is indicated by the red arc, and the deeper orange and pale orange arcs represent the N50 and N90 lengths. A light grey spiral at the centre shows the cumulative scaffold count on a logarithmic scale. A summary of complete, fragmented, duplicated, and missing BUSCO genes in the hymenoptera_odb10 set is presented at the top right. An interactive version of this figure can be accessed on the BlobToolKit viewer.

**Figure 6.**
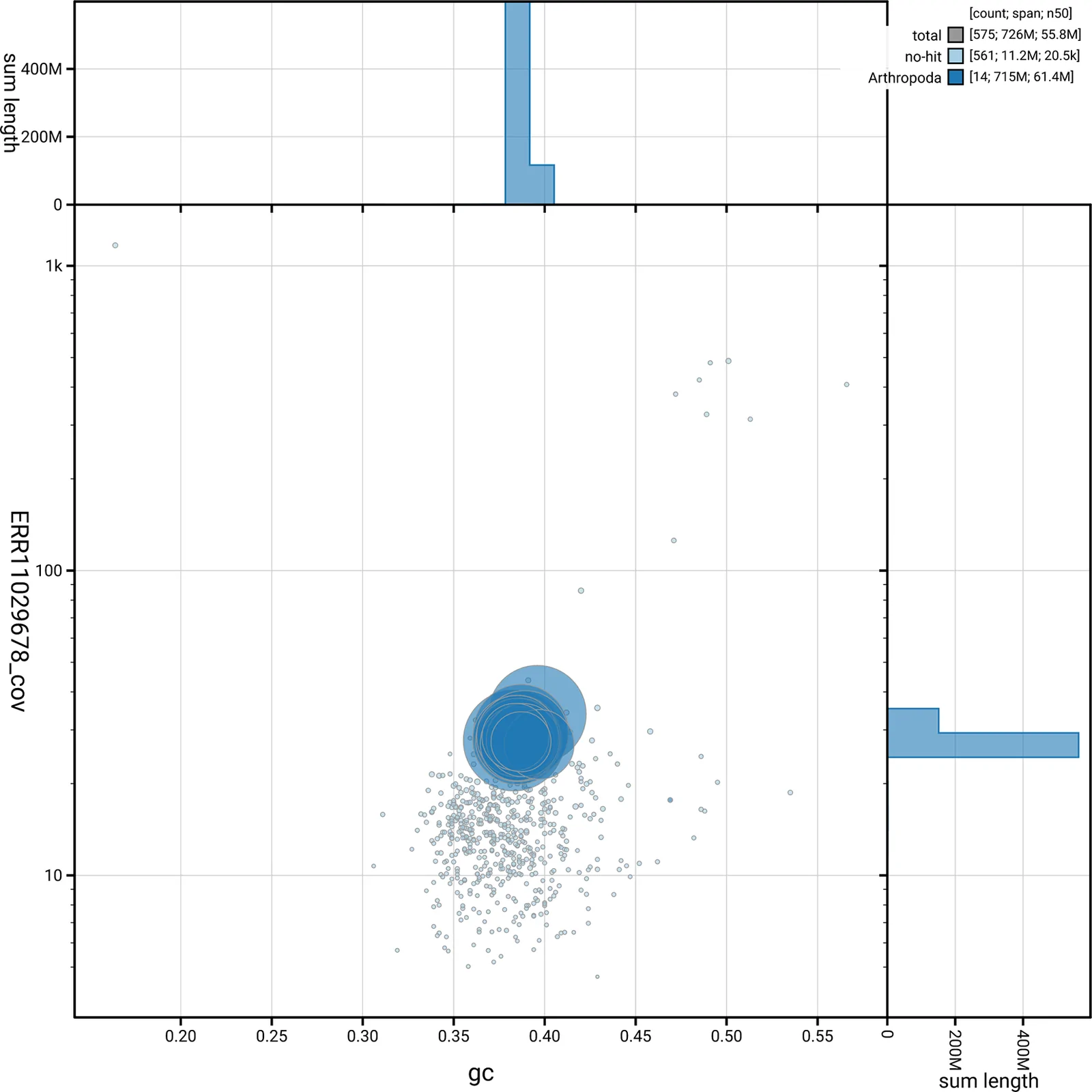
BlobToolKit blob plot for iyOphCras1.1. The plot shows base coverage (vertical axis) and GC content (horizontal axis). The circles represent scaffolds, with the size proportional to scaffold length and the colour representing phylum membership. The histograms along the axes display the total length of sequences distributed across different levels of coverage and GC content. An interactive version of this figure is available on the BlobToolKit viewer.

BUSCO v.6.0.0 analysis using the hymenoptera_odb10 reference set ( *n* = 5 991) identified 95.7% of the expected gene set (single = 95.2%, duplicated = 0.5%). The snail plot in Figure 5 summarises the scaffold length distribution and other assembly statistics for the primary assembly. The blob plot in Figure 6 shows the distribution of scaffolds by GC proportion and coverage.

Table 4 lists the assembly metric benchmarks adapted from Rhie *et al.* (2021) and the Earth BioGenome Project Report on Assembly Standards. The EBP metric, calculated for the primary assembly, is **5.C.Q54**.

**Table 4.** Earth Biogenome Project summary metrics for the *Ophion crassicornis* assembly.

Measure	Value	Benchmark
EBP summary (primary)	5.C.Q54	6.C.Q40
Contig N50 length	0.37 Mb	≥ 1 Mb
Scaffold N50 length	55.84 Mb	= chromosome N50
Consensus quality (QV)	Primary: 54.8; alternate: 56.5; combined: 55.8	≥ 40
*k*-mer completeness	Primary: 89.10%; alternate: 82.05%; combined: 98.11%	≥ 95%
BUSCO	C:95.7% [S:95.2%, D:0.5%], F:0.9%, M:3.5%, n:5 991	S > 90%; D < 5%
Percentage of assembly assigned to chromosomes	98.51%	≥ 90%

*Note:* The EBP summary uses log10(Contig N50); chromosome-level (C) or log10(Scaffold N50); Q (Merqury QV). BUSCO: C = complete; S = single-copy; D = duplicated; F = fragmented; M = missing; n = orthologues.

**Table 5.** Software versions and sources used for *Ophion crassicornis.*

Software	Version	Source
BLAST	2.14.0	ftp://ftp.ncbi.nlm.nih.gov/blast/executables/blast+/
BlobToolKit	4.4.6	https://github.com/blobtoolkit/blobtoolkit
BUSCO	6.0.0	https://gitlab.com/ezlab/busco
bwa-mem2	2.2.1	https://github.com/bwa-mem2/bwa-mem2
DIAMOND	2.1.8	https://github.com/bbuchfink/diamond
fasta_windows	0.2.4	https://github.com/tolkit/fasta_windows
FastK	1.1	https://github.com/thegenemyers/FASTK
GenomeScope2.0	2.0.1	https://github.com/tbenavi1/genomescope2.0
Gfastats	1.3.6	https://github.com/vgl-hub/gfastats
Hifiasm	0.16.1	https://github.com/chhylp123/hifiasm
HiGlass	1.13.4	https://github.com/higlass/higlass
MerquryFK	1.1.0-c1	https://github.com/thegenemyers/MERQURY.FK
Minimap2	2.28-r1209	https://github.com/lh3/minimap2
Oatk	1.alpha	https://github.com/c-zhou/oatk
MultiQC	1.14; 1.17 and 1.18	https://github.com/MultiQC/MultiQC
Nextflow	24.10.4	https://github.com/nextflow-io/nextflow
PretextSnapshot	0.0.5	https://github.com/sanger-tol/PretextSnapshot
PretextView	1.0.3	https://github.com/sanger-tol/PretextView
purge_dups	1.2.3	https://github.com/dfguan/purge_dups
samtools	1.2; 1.21	https://github.com/samtools/samtools
sanger-tol/ascc	0.1.0	https://github.com/sanger-tol/ascc
sanger-tol/blobtoolkit	v0.9.0	https://github.com/sanger-tol/blobtoolkit
sanger-tol/curationpretext	1.4.2	https://github.com/sanger-tol/curationpretext
Seqtk	1.3	https://github.com/lh3/seqtk
Singularity	3.9.0	https://github.com/sylabs/singularity
TreeVal	1.4.0	https://github.com/sanger-tol/treeval
YaHS	1.1a.2	https://github.com/c-zhou/yahs

## Author information

Contributors are listed at the following links:

• Members of the Natural History Museum Genome Acquisition Lab
• Members of the Darwin Tree of Life Barcoding collective
• Members of the Wellcome Sanger Institute Tree of Life Management, Samples and Laboratory team
• Members of Wellcome Sanger Institute Scientific Operations – Sequencing Operations
• Members of the Wellcome Sanger Institute Tree of Life Core Informatics team
• Members of the Tree of Life Core Informatics collective
• Members of the Darwin Tree of Life Consortium

## Wellcome sanger institute – legal and governance

The materials that have contributed to this genome note have been supplied by a Darwin Tree of Life Partner. The submission of materials by a Darwin Tree of Life Partner is subject to the **‘Darwin Tree of Life Project Sampling Code of Practice’**, which can be found in full on the Darwin Tree of Life website. By agreeing with and signing up to the Sampling Code of Practice, the Darwin Tree of Life Partner agrees they will meet the legal and ethical requirements and standards set out within this document in respect of all samples acquired for, and supplied to, the Darwin Tree of Life Project. Further, the Wellcome Sanger Institute employs a process whereby due diligence is carried out proportionate to the nature of the materials themselves, and the circumstances under which they have been/are to be collected and provided for use. The purpose of this is to address and mitigate any potential legal and/or ethical implications of receipt and use of the materials as part of the research project, and to ensure that in doing so we align with best practice wherever possible. The overarching areas of consideration are:

• Ethical review of provenance and sourcing of the material
• Legality of collection, transfer and use (national and international)

Each transfer of samples is further undertaken according to a Research Collaboration Agreement or Material Transfer Agreement entered into by the Darwin Tree of Life Partner, Genome Research Limited (operating as the Wellcome Sanger Institute), and in some circumstances, other Darwin Tree of Life collaborators.

## Data Availability

European Nucleotide Archive: Ophion crassicornis. Accession number PRJEB60661; https://identifiers.org/ena.embl/PRJEB60661. The genome sequence is released openly for reuse. The *Ophion crassicornis* genome sequencing initiative is part of the Darwin Tree of Life Project (PRJEB40665) and the Sanger Institute Tree of Life Programme (PRJEB43745). All raw sequence data and the assembly have been deposited in INSDC databases. The genome will be annotated using available RNA-Seq data and presented through the Ensembl pipeline at the European Bioinformatics Institute. Raw data and assembly accession identifiers are reported in Tables 1 and 2. Production code used in genome assembly at the WSI Tree of Life is available at https://github.com/sanger-tol . Table 5 lists software versions used in this study.
