# Supplementary material for: The genome sequence of an ichneumonid wasp, Ophion crassicornis Brock, 1982 (Hymenoptera: Ichneumonidae)
Source: Wellcome Open Res. 2026 Jul 27;11:467. [Version 1] doi: 10.12688/wellcomeopenres.26848.1 (PMC13562945; doi:10.12688/wellcomeopenres.26848.1)
Supplement: Peer review history

# Reviewer report: Reviewer response for version 1

**DOI:** 10.21956/wellcomeopenres.29563.r165298

**Published:** 2026-09-08

**Authors:** Rajib Deb

**Affiliations:** 1 ICAR-National Research Centre, Guwahat, Assam, India

This Data Note presents a chromosome-level genome assembly of the parasitoid wasp *Ophion crassicornis* generated as part of the Darwin Tree of Life project. Together with a mitochondrial genome, the work offers a 726.34 Mb genome assembly, of which 13 chromosome-level pseudomolecules account for 98.51% of the sequence. One of the work's main strengths is the utilization of PacBio HiFi, Hi-C, and RNA-seq data along with thorough assembly curation and quality assessment.

 My concerns are as below

 1. The genome was generated from a single female specimen, which is appropriate for establishing a reference genome but limits assessment of within-species genomic variation and population-level diversity. This limitation should be explicitly acknowledged, particularly given the taxonomic complexity within *Ophion*.

 2.Although the chromosome-level scaffolding is excellent, the contig N50 is only 0.37 Mb, substantially below the ≥1 Mb EBP benchmark. The authors should briefly discuss the implications of this relatively fragmented contig-level assembly and explain how the extensive Hi-C scaffolding compensates for this limitation.

 3.The manuscript currently focuses primarily on genome assembly and quality assessment. Since RNA-seq data have already been generated, the authors should clarify the intended role and future availability of gene annotation and explain how the reference genome will facilitate comparative genomics, functional studies and investigation of the biology of *O. crassicornis*. The manuscript currently indicates that annotation will be undertaken through the Ensembl pipeline.

 4. RNA-seq was generated from the same individual used for genome sequencing and from abdominal tissue. Therefore, these data provide useful transcriptomic support for future gene annotation but cannot provide biological or population-level transcriptomic validation. This distinction should be stated clearly.

 5. The authors should strengthen the concluding discussion by briefly highlighting how this reference genome can support comparative genomic analyses, species delimitation, parasitoid biology and future studies of host–parasitoid interactions, while avoiding claims beyond the scope of the present dataset.

Are sufficient details of methods and materials provided to allow replication by others?

Partly

Is the rationale for creating the dataset(s) clearly described?

Partly

Are the datasets clearly presented in a useable and accessible format?

Partly

Are the protocols appropriate and is the work technically sound?

Partly

Reviewer Expertise:

Genomics

I confirm that I have read this submission and believe that I have an appropriate level of expertise to confirm that it is of an acceptable scientific standard, however I have significant reservations, as outlined above.

# Reviewer report: Reviewer response for version 1

**DOI:** 10.21956/wellcomeopenres.29563.r165306

**Published:** 2026-09-04

**Authors:** Peng-Fei Ma

**Affiliations:** 1 Chinese Academy of Sciences, Kunming, China

Here the complete genome of Ophion crassicornis was sequenced and assembled. A total length of 726.34 Mb sequences were presented, with 98.5% of them scaffolded into 13 chromosomal pseudomolecules. Evaluation of the assembly, from BUSCO to k-mer completeness, all revealed a high-quality reference genome obtained. in addition, the mitochondrial genome was also assembled. The newly generated genome will be valuable genetic resource for comparative analyses of Ophion species in future.

Are sufficient details of methods and materials provided to allow replication by others?

Yes

Is the rationale for creating the dataset(s) clearly described?

Yes

Are the datasets clearly presented in a useable and accessible format?

Yes

Are the protocols appropriate and is the work technically sound?

Yes

Reviewer Expertise:

Plant genomics and evolution

I confirm that I have read this submission and believe that I have an appropriate level of expertise to confirm that it is of an acceptable scientific standard.

# Reviewer report: Reviewer response for version 1

**DOI:** 10.21956/wellcomeopenres.29563.r163725

**Published:** 2026-08-20

**Authors:** Jurate De Prins

**Affiliations:** 1 Flemish Entomological Society, Vrieselhof, Belgium

I appreciate very much that the taxonomic species presentation is within the broader context of the knowledge of how many species are in the UK belonging to the same genus, how rare or common they are, how they are distributed and which species of Noctuidae (Lepidoptera) are the hosts. Also, the knowledge gaps are indicated. This is a very positive development.  In several of my reviews for Wellcome Sanger Institute articles, I indicated that correct species ID is crucial for any further work and that accompanying taxonomic information is needed. Happy that my comments are taken into account. 

 In this article, I would recommend presenting the reader with slightly broader information on genetic aspects: is a 13-chromosome set a usual chromosome number for Ichneumonidae? How about the sex determination mechanism and sex chromosomes? Are they the same as autosomes or different? As far as I remember from my studies many years ago, Ichneumonidae possess gigantic chromosomes. Does this research confirm that, and if so, then how much bigger are they in comparison with other insect groups?

 One more positive aspect I would like to stress is that the specimen studied was taken from the collection and captured five years ago. It would be interesting to know how old the specimens can be to be processed by the modern molecular techniques.

Are sufficient details of methods and materials provided to allow replication by others?

Yes

Is the rationale for creating the dataset(s) clearly described?

Yes

Are the datasets clearly presented in a useable and accessible format?

Yes

Are the protocols appropriate and is the work technically sound?

Yes

Reviewer Expertise:

Taxonomy, Karyology, Biodiversity informatics

I confirm that I have read this submission and believe that I have an appropriate level of expertise to confirm that it is of an acceptable scientific standard.
